# Supplementary material for: Prevalence of Acinetobacter baumannii Multidrug Resistance in University Hospital Environment
Source: Antibiotics (Basel). 2025 May 10;14(5):490. doi: 10.3390/antibiotics14050490 (PMC12108267; doi:10.3390/antibiotics14050490)

Table S1 Trends in the percentage of *A. baumannii* resistance to various antimicrobials from 2018 to 2023, a period that began with the introduction of Antimicrobial Diagnostic Stewardship in our hospital.

Antimicrobials	2018	2019	2020	2021	2022	2023	P value
Meropenem	100%	100%	89.7%	94.7%	97.7%	94.4%	0.42
Ciprofloxacine	100%	100%	97.8%	94.7%	97.7%	94.4%	0.04
Levofloxacine	100%	100%	91.1%	94.7%	97.5%	94.4%	0.33
Amikacine	100%	97.3%	81.8%	94.7%	68.2%	94.4%	0.36
Gentamicine	100%	97.5%	87.2%	94.7%	70.4%	83.3%	0.07
Tobramicine	100%	97.5%	87.2%	94.7%	79.5%	77.7%	0.42
Colistine	4.1%	0%	2.1%	5.2%	0%	0%	0.42
TMP-SMX	95.8%	92.3%	85.1%	94.7%	68.1%	100%	0.71
Cefiderocol	*	*	*	0%	0%	0%	//

Table S2 Trends in the percentage of MDR *A. baumannii* from 2018 to 2023

MDR	2018	2019	2020	2021	2022	2023	P value
Urine	100%	100%	92.3%	//	33.3%	100%	0.48
Blood	100%	100%	95.6%	87.5%	60%	90.9%	0.19
Pus	100%	95.6%	91.6%	100%	77.8%	100%	0.59
Total	100%	97.4%	93.7%	94.7%	63.6%	94.4%	0.30

Figure S1 Differences in percentage of MDR *A. baumannii*

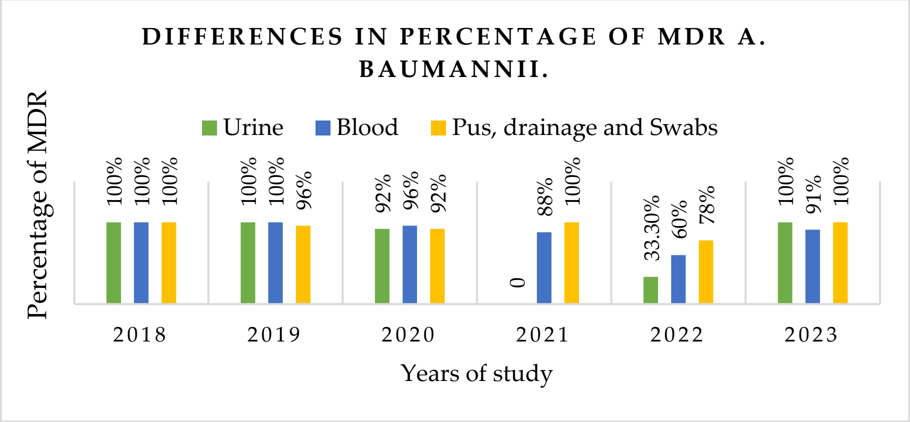

Supplement: Supplementary file 1 [file antibiotics-14-00490-s001.zip › antibiotics-3584927-supplementary.pdf]
